# Emotional and musical factors combined with song-specific age predict the subjective autobiographical saliency of music in older adults

**DOI:** 10.1177/03057356231186961

**Published:** 2023-10-16

**Authors:** Ilja Salakka, Anni Pitkäniemi, Emmi Pentikäinen, Pasi Saari, Petri Toiviainen, Teppo Särkämö

**Affiliations:** 1 Music, Ageing and Rehabilitation Team, Cognitive Brain Research Unit, Department of Psychology and Logopedics, Faculty of Medicine, University of Helsinki, Helsinki, Finland; 2 Centre of Excellence in Music, Mind, Body and Brain, University of Jyväskylä and University of Helsinki, Helsinki, Finland; 3 Rehabilitation Foundation, Helsinki, Finland; 4 Department of Music, Art and Culture Studies, University of Jyväskylä, Jyväskylä, Finland

**Keywords:** emotion, memory, aging, rhythm, timbre, tonality, modeling

## Abstract

Music that evokes strong emotional responses is often experienced as autobiographically salient. Through emotional experience, the musical features of songs could also contribute to their subjective autobiographical saliency. Songs which have been popular during adolescence or young adulthood (ages 10–30) are more likely to evoke stronger memories, a phenomenon known as a reminiscence bump. In the present study, we sought to determine how song-specific age, emotional responsiveness to music, musical features, and subjective memory functioning contribute to the subjective autobiographical saliency of music in older adults. In a music listening study, 112 participants rated excerpts of popular songs from the 1950s to the 1980s for autobiographical saliency. Additionally, they filled out questionnaires about emotional responsiveness to music and subjective memory functioning. The song excerpts’ musical features were extracted computationally using MIRtoolbox. Results showed that autobiographical saliency was best predicted by song-specific age and emotional responsiveness to music and musical features. Newer songs that were more similar in rhythm to older songs were also rated higher in autobiographical saliency. Overall, this study contributes to autobiographical memory research by uncovering a set of factors affecting the subjective autobiographical saliency of music.

Memories are important for the sense of self, and music is an exceptionally powerful stimulus for evoking them, even in people with dementia (Cuddy et al., 2015). Because music tends to be personally salient to most people, it is often used for emotional self-regulation throughout adulthood (Saarikallio, 2010). That being said, strong emotional responses to music are often connected to strong musical memories (Janata et al., 2007; Salakka et al., 2021; Schulkind et al., 1999; Schulkind & Woldorf, 2005) and they can serve as a strengthening factor in episodic memory encoding (Mado Proverbio et al., 2015). Musical memory formation has been found to be intimately linked to the experience of reward from music, modulated by the dopaminergic mesolimbic system (Ferreri et al., 2021, 2019), which can, at least partially, explain this strong connection between musical emotions and memories. Conversely, when a repeatedly heard song has become familiar, it tends to be experienced as higher in emotional intensity and valence (Ali & Peynirciogˇgˇlu, 2010; Peretz et al., 1998). In general, these findings suggest that both the consolidation of new musical memories and the recall of old musical memories are strongly driven by emotions. Still, individual differences in emotional responsiveness to music affect what kind of emotional reaction music is likely to evoke (Juslin et al., 2022); therefore, these differences might also play a part in the subjective autobiographical saliency of music.

Musical memories are not static throughout the life course. In younger people, they more typically resemble specific life events, whereas in older people, they tend to be more affectively focused and engage dorsomedial prefrontal regions (Ford et al., 2016), which are known to both track the tonal structure of music and encode its emotional and autobiographical saliency (Janata, 2009; Janata et al., 2002). Importantly, musical memories can often be effectively retrieved even in neurological disorders in which memory retrieval is typically strongly impaired, such as in Alzheimer’s disease (El Haj, Fasotti, & Allain, 2012; El Haj, Postal, & Allain, 2012). Generally, older adults tend to remember memories better from their youth when compared to newer memories (very recent memories excluded) or memories from their first 10 years. Known as a reminiscence bump, this is a robust cognitive phenomenon found in adults across different ages (Janssen et al., 2005) and also cross-culturally (Conway et al., 2005). It is characterized by an increased ease of retrieval of memories from age 10 to 30, usually peaking somewhere between 13 and 18 years on average (Janssen et al., 2005). However, the estimated age range for the reminiscence bump varies to some extent depending on the method used to activate the memories (Munawar et al., 2018). The reminiscence bump has also been observed in the context of musical memories, typically occurring between 5 and 24 years and peaking somewhere around 15 years (Jakubowski et al., 2020; Platz et al., 2015; Schulkind et al., 1999; Schulkind & Woldorf, 2005). Interestingly, Krumhansl and Zupnick (2013) found evidence for the cascading reminiscence bump, meaning that people show a bump in autobiographical memory recall for the songs their parents listened to as young adults, because as children they were exposed to those songs in their home environment.

Although musical memories can usually be recalled relatively well even under neurodegenerative conditions (Cuddy & Duffin, 2005; Cuddy et al., 2012; El Haj, Postal, & Allain, 2012; Vanstone & Cuddy, 2009b), general subjective memory changes also become more common during healthy aging. While subjective memory difficulties do not necessarily reflect a concurrent decline in objective memory performance, they are sometimes related to deterioration in other cognitive domains, such as slower processing speed (Vaskivuo et al., 2018), and may precede objective memory problems (Reid & MacLullich, 2006), and even dementia (Schmand et al., 1996). Regular music listening has been observed to improve subjective memory in older adults experiencing early signs of cognitive decline (Innes et al., 2017), suggesting a relationship between music and general memory. However, it is still unknown if subjective memory difficulties are related to the autobiographical saliency of musical memories.

While the emotional experience of music plays a central part in the formation and retrieval of musical memories, musical features might also independently contribute to the subjective autobiographical saliency of a song. Broadly comprised of tonal, rhythmic, and timbral aspects, music can be viewed as structured sound. It can be broken down into smaller sources of information, such as spectral properties of musical signals, hereafter referred to as musical features. Music Information Retrieval (MIR) methods have made it possible to extract musical features related to basic musical elements such as timbre, rhythm, and tonality from the audio signal, which in turn can be used in statistical analyses exploring their relationship to the subjective experience of music. These computationally extracted musical features have previously been successfully used to predict both the subjective emotional experience (Gingras et al., 2014; Schubert, 2004; Singer et al., 2016) and the autobiographical memories evoked by a song (Salakka et al., 2021). Using MIR, Salakka et al. (2021) reported that the salience of the musical pulse, the relative strength of high harmonics, and fluctuation in frequencies between 200 and 800 Hz predicted both music-evoked emotions and memories in older adults. The results of these previous studies leave us with an open question of whether musical features may individually contribute to the autobiographical saliency of a song after accounting for age and emotional responsiveness to music.

Taken together, many different factors related to the listener or to the music itself potentially underlie the autobiographical saliency of a song. In the present study, our focus was to identify if some of these, namely song-specific age (SSA, the age of the listener when the song was popular), emotional responsiveness to music, subjective memory functioning, and musical features, have an effect on the overall autobiographical saliency of music. Concerning musical features, we took into account some of the fundamental elements of music: tonality, rhythm, and timbre. Salakka et al. (2021) proposed that because older music, compared to more contemporary music, tends to have a different kind of musical “profile” (i.e. differences in musical features), people who grew up listening to that kind of music may find similar features more appealing also in newer music. Therefore, we expected to see that in the group of newer songs, those having similar musical profiles with older songs (measured with MIR scores) would be experienced as more autobiographically salient.

## Methods

### Participants

The participants for the listening experiment were 112 healthy older adults, 86 women and 26 men, aged 60–86 years (*M* *=* 70.7, *SD* = 5.4), with a mean education level of 4.8 (*SD* = 2.0, range = 1–8) on the International Standard Classification of Education (ISCED) 8-point scale. All were from the Helsinki metropolitan area and were participating in an ongoing study on the neurocognitive effects of senior choir singing (78 participants were amateur choir singers, and 35 were nonsingers). The possible responses when asking about gender were “Man,” “Woman,” and “Other.” The participants were recruited from the Adult Education Centers of the Cities of Helsinki, Espoo, and Vantaa and from different senior citizens’ associations and independent choirs through presentations, flyers, and email advertisements. All participants were Finnish-speaking and had no history of neurological (e.g. dementia, stroke) or severe psychiatric (e.g. schizophrenia, bipolar disorder) disorders. The study was approved by the Ethical Review Board in the Humanities and Social and Behavioral Science of the University of Helsinki, and all participants gave written informed consent.

### Music listening experiments and questionnaires

The participants performed an old-time music rating task (OMRT) consisting of 70 song excerpts (see “Stimuli and musical feature extraction” section). A web application for OMRT was developed specifically according to the needs of this study. The Finnish company Sentina Ltd was responsible for the technical implementation of the application and data reporting system, which ran on Sentina’s cloud service. The participants rated each of the song excerpts by answering five questions assessing experienced valence, emotional intensity, arousal, familiarity, and the autobiographical saliency of the song (5-point Likert scale). In the present study, we used only autobiographical saliency as a dependent variable on prediction models. It was measured with a question, “Kuinka paljon omakohtaisia muistoja kappale sinussa herätti?” which translates directly as “How much did the song evoke personal memories?” The rating was from “ei lainkaan (none at all)” (1) to “erittäin paljon (very much)” (5). Even though the translation sounds somewhat quantitative in nature, the Finnish phrasing has a connotation not just to the number of memories but also to their strength or intensity, indicating how “personally memory-evoking” the song was. The answering format was a forced choice, and questions were presented one at a time. When answering questions, the participants were able to listen to the song excerpt again as many times as they wanted. For each song, the interface also allowed the participants to share any memories related to the song by writing text or recording audio (this qualitative data is not part of this study). The participants were able to do the whole listening experiment in their preferred place (e.g. at home), using their own computer or a tablet computer which was lent and set up by the researchers. The use of headphones or external speakers was recommended, and the participants were asked to set the volume to a comfortable but clearly audible level. The experiment started with a short practice session to familiarize the participants with the basic functionality of the interface. The whole experiment took 2.5 hr to complete on average.

The participants self-assessed their subjective memory by filling out the Prospective-Retrospective Memory Questionnaire (PRMQ; Smith et al., 2000). In this study, we used a PRMQ total score as a measure of subjective memory (*n* = 112, *M* *=* 31.08, *SD* = 7.50, range = 16–52). The participants also filled out the Musical Engagement Questionnaire (MusEQ; Vanstone et al., 2016) to self-assess their engagement with music in daily life. We decided a priori to use only the Emotion subscale of the MusEQ (*n* = 111, *M* *=* 3.60, *SD* = 0.65, range = 1–5) in this study to assess the relationship between participants’ emotional responsiveness to music and their ratings of the autobiographical saliency of music.

### Stimuli and musical feature extraction

The song pool consisted of 140 songs in total divided into two lists (A and B, 70 songs in each), matched for song genre and era. Each list comprised 10 folk songs plus 15 songs from each of the four decades from the 1950s to the 1980s, representing different musical genres (popular, rock, and jazz) and languages (Finnish or English). This time period for stimulus songs was selected because it was optimal for evoking autobiographical memories in the participants of the present study (Jakubowski et al., 2020; Janssen et al., 2005; Platz et al., 2015; Schulkind et al., 1999; Schulkind & Woldorf, 2005). The participants were allocated into two groups (corresponding to A and B song lists) matching for age, gender, and choir singing background. There was no notable difference in the autobiographical saliency ratings between the groups, Akaike information criterion (AIC) 24,756 -> 24,755; χ^2^(1) = 3.506, *p* *=* .061. The full song list with a more detailed description of forming it is presented in Supplemental Material 1. For each song, an excerpt of the most characteristic and well-known part (e.g. the chorus) was selected. The average length of the excerpts was 30 s (range = 18–37 s), and half sine wave fade-ins (1 s) and fade-outs (3 s) were added to each excerpt to make the listening experience smooth. The MP3 format was used for all audio files.

Concerning the selection of musical features, many studies have used a principal component analysis (PCA) approach to combine many features into fewer components. For example, Salakka et al. (2021) used a set of 24 musical features and applied the PCA, resulting in a six-component solution. However, none of the components had a clear tonal interpretation despite some tonal features in the original set. In some other studies utilizing the PCA or similar procedures, pulse clarity and key clarity have had strong loadings in their own distinct components, being also the only components that do not represent timbre in any way (Alluri et al., 2012; Burunat et al., 2016; Toiviainen et al., 2020). In the present study, we wanted to emphasize the interpretability of the features, so we decided to use pulse clarity and key clarity to represent rhythm and tonality. Temporal and spectral clarity has also led to better recognition of song material (Albouy et al., 2020), which further supports using clarity-based features for these domains. For timbre, we chose spectral centroid to be the representing feature, which has previously been found to characterize fundamental aspects of timbre, such as brightness (Alluri et al., 2012; McAdams et al., 1995; Salakka et al., 2021). In this way, we formed a set of three musical features, representing tonality (key clarity), rhythm (pulse clarity), and timbre (spectral centroid), with clear interpretations to use in the present study. Supplemental Material 3 shows all three musical features plotted against the year in which the song was popular. All musical features were extracted with MIRtoolbox 1.7 (Lartillot & Toiviainen, 2007) on MATLAB R2018a using a default sampling rate (44,100 Hz, typical for MP3 files). Frame lengths of 0.025 sec (for spectral centroid) and 3 sec (for pulse clarity and key clarity) were used with a frame overlap of 50%. The mean for each musical feature (computed across the frames) was used in the statistical analyses. Because of the large skewness in pulse clarity, logarithmic transformation was applied to avoid biasing effect on the results (skewness = 0.94–> 0.37).

### Statistical analyses

Statistical analyses were conducted with R-language version 4.0.4 (R Core Team, 2021) in RStudio environment version 1.4.1717. Linear mixed-effects models (LMM) were applied to fit models predicting the autobiographical saliency of the songs using the R package *lme4* (Bates et al., 2015). LMM was used in this study to account for the structure of the data (two different song lists with different participants) and for individual variance in the autobiographical saliency ratings. The fit of the models was assessed using marginal (*R*^2^m) and conditional (*R*^2^c) *R*^2^ values for LMM (Nakagawa & Schielzeth, 2013) computed with the R package *MuMIn* (Barton, 2020), and AIC and χ^2^ values from the core R packages (R Core Team, 2021). Multicollinearities in LMM predictors were assessed by inspecting VIF values using the R package *car* (Fox & Weisberg, 2019). Residuals of the models were examined visually using the core R packages (R Core Team, 2021). Differences and interactions between SSA (low ⩽ 20 years/high > 20 years) and autobiographical saliency (low score ⩽ 3/high score > 3) groups in the domains of the musical features were assessed with LMM. All graphical plots were created with *ggplot2*-package (Wickham, 2016). Where comparison between different predictors was needed, the standardization of the predictor variables was done by subtracting the mean and dividing by the standard deviation for each variable. This computation resulted in scaled variables having a mean of 0 and a standard deviation of 1. The SSA was calculated as the difference between the year when the song was popular in the Finnish music charts and the birth year of the participant. For the purposes of this paper, folk songs were removed from the data because computing their SSA is ambiguous.

## Results

Here, we present three sets of LMM analyses to predict autobiographical saliency. First, we inspect the relationship between SSA and the autobiographical saliency accounting for the reminiscence bump. Second, we present five models with multiple predictors (subjective emotional response to music, subjective memory functioning, musical features, SSA, and age and gender as demographic covariates) to inspect their individual predictive ability after accounting for others. Last, we present three models in which we examine the interactions between SSA and autobiographical saliency in different musical domains.

### The relationship between SSA and the autobiographical saliency

**Figure 1.**
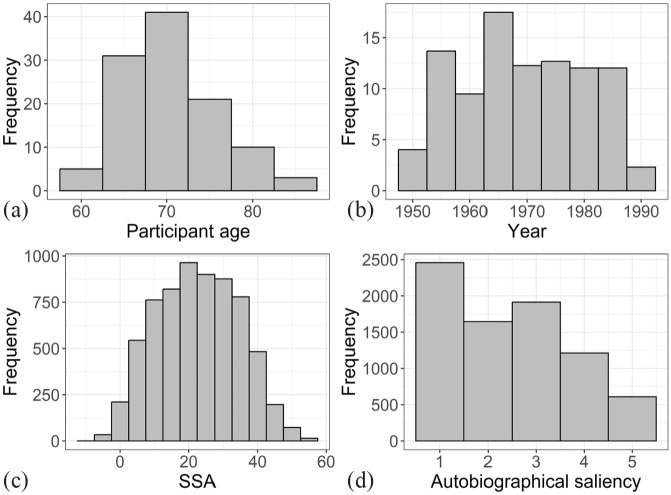
Distributions of (a) Participant Age (*M* *=* 70.7, *SD* = 5.4), (b) The Year the Song was Popular in Finnish Radio Channels (*M* *=* 1,969, *SD* = 10.7), (c) SSA (*M* *=* 22.6, *SD* = 12.0), and (d) The Autobiographical Saliency (*M* *=* 2.5, *SD* = 1.3).

**Figure 2.**
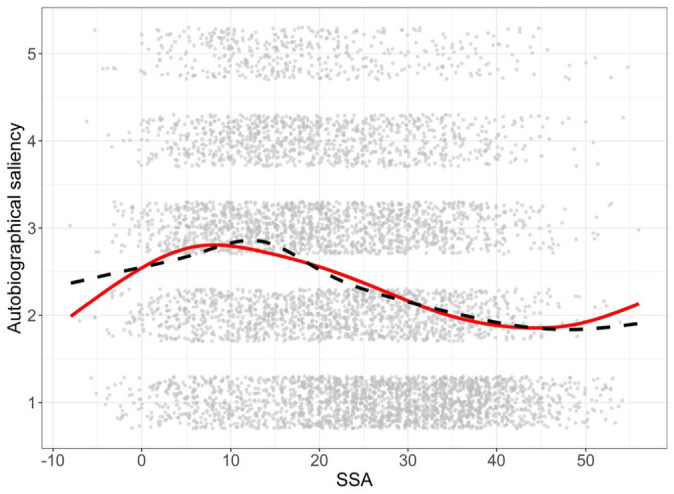
The Cubic Function (Smooth Line) Barely Misses the Peak and the Very Ends of the LOESS Curve (Dashed Line) but Otherwise Fits Well With the Reminiscence Bump Phenomenon in the Present Data.

Along with the participant’s age and the year the song was popular on Finnish radio channels, Figure 1 provides distributions for SSA and autobiographical saliency. To examine the reminiscence bump, we plotted the autobiographical saliency against SSA. Visual inspection of the LOESS curve (locally estimated scatterplot smoothing) shows a clear bump around the teenage years, peaking at the age of 11 (Figure 2, dashed line). Because of this nonlinear relationship between the SSA and the autobiographical saliency, it was possible that a simple linear function was not the most appropriate model to use here. Thus, we decided to fit higher order SSA terms to the data to see if they do better in accounting for the reminiscence bump phenomenon than a simple linear function. As can be seen from Figure 1 and Table 1, the cubic function fits quite well to the LOESS curve, providing a more parsimonious fit over the quadratic and linear functions. The quartic function does not increase the fit of the model enough compared to the cubic function but makes the model more complex and runs into the danger of overfitting. Based on these examinations, we used the cubic SSA model as a predictor in the next set of LMM analyses by including all relevant SSA terms to the models.

**Table 1.** Comparing Different Models Predicting the Autobiographical Saliency by Akaike Information Criterion and χ^2^-test.

	AIC	χ^2^	*p* value
Linear^a^	20,430	11.859	.0006
Quadratic^b^	20,420		
Quadratic^b^	20,420	81.48	<.0001
Cubic^c^	20,341		
Cubic^c^	20,341	0.6807	.4094
Quartic^d^	20,342		

*Note*. AIC: Akaike information criterion.

a SSA + ID_random_, *R*^2^m = 0.0537, *R*^2^c = 0.2595.

b SSA + SSA^2^ + ID_random_, *R*^2^m = 0.0558, *R*^2^c = 0.2608.

c SSA + SSA^2^ + SSA^3^ + ID_random_, *R*^2^m = 0.0604, *R*^2^c = 0.2678.

d SSA + SSA^2^ + SSA^3^ + SSA^4^ + ID_random_, *R*^2^m = 0.0605, *R*^2^c = 0.2678.

### Step-by-step models predicting the autobiographical saliency

We built five LMMs step-by-step by adding new variable(s) on top of the others to see how much they improved the model (in terms of *R*^2^m/*R*^2^c and AIC) and what contribution each of the predictors had beside others (Table 2; VIF values presented in Supplemental Material 2). Age and gender (Model 1) and subjective memory functioning (PRMQ; Model 2) had no explanatory power for autobiographical saliency. Subjective emotional responsiveness to music (Model 3) significantly predicted the autobiographical saliency after adjusting for Model 2 effects. This is seen as a decrease in the between-participants variance, arising from the fact that emotional responsiveness reflects individual differences between participants. Model 4 added the cubic SSA effect to the model. Under severe, but inevitable, multicollinearity between different SSA terms, their beta coefficients cannot be reliably compared to other terms in the model. This, however, does not affect their added predictive value in the model. Compared to Model 3, Model 4 increased *R*^2^ values by over 6% and decreased residual variance by ~10%, indicating that SSA is the best single predictor of autobiographical saliency among the variables used here. In the full model (Model 5), musical features were added, increasing the marginal *R*^2^ to almost 11%. All three musical features (pulse clarity, key clarity, and spectral centroid) had a significant statistical effect. Of the musical features, pulse clarity was the best predictor of autobiographical saliency, while the effects of key clarity and spectral centroid were clearly smaller. For both models (4 and 5), the effects were seen only in the residual variance, reflecting average consistency across the participants in these matters.

**Table 2.** Step-by-Step Models Predicting the Autobiographical Saliency.

Fixed effects	Null model	Model 1	Model 2	Model 3	Model 4	Model 5
	β [95% CI]	β [95% CI]	β [95% CI]	β [95% CI]	β [95% CI]	β [95% CI]
Intercept*	2.472 [2.362, 2.584]	2.468 [2.358, 2.578]	2.467 [2.358, 2.577]	2.468 [2.363, 2.572]	2.396 [2.290, 2.501]	2.440 [2.335, 2.545]
Age		−0.091 [−0.038, 0.004]	−0.098 [−0.208, 0.013]	−0.070 [−0.177, 0.037]	0.0002 [−0.1085, 0.1091]	−0.062 [−0.171, 0.046]
Gender		0.005 [−0.245, 0.268]	0.009 [−0.101, 0.119]	0.037 [−0.069, 0.143]	0.050 [−0.057, 0.157]	0.051 [−0.056, 0.157]
PRMQ			0.045 [−0.066, 0.156]	0.015 [−0.093, 0.122]	0.002 [−0.106, 0.110]	0.001 [−0.106, 0.109]
Emotion				**0.179** [0.070, 0.288]	**0.195** [0.085, 0.304]	**0.194** [0.085, 0.303]
SSA					**0.682** [0.485, 0.888]	**0.693** [0.491, 0.896]
SSA^2^					−**2.220** [−2.673, −1.766]	−**1.886** [−2.339, −1.432]
SSA^3^					**1.306** [1.023, 1.588]	**1.108** [0.827, 1.388]
Spectral centroid						−**0.076** [−0.110, −0.043]
Key clarity						**0.034** [0.007, 0.062]
Pulse clarity						−**0.206** [−0.242, −0.170]
Random effects	Variance	Variance	Variance	Variance	Variance	Variance
ID (Intercept)	0.336	0.334	0.335	0.308	0.309	0.308
Residual	1.320	1.314	1.314	1.314	1.182	1.142
PCV (ID)		0.60%	−0.30%	8.06%	−0.32%	0.32%
PCV (Residual)		0.45%	~0%	~0%	10.04%	3.38%
*R*^2^m		0.50%	0.65%	2.41%	8.50%	10.92%
*R*^2^c		20.67%	20.83%	20.92%	27.49%	29.83%
AIC	24,760.35	24,515.37	24,520.64	24,516.81	20,369.21	20,170.14
χ^2^ (*p* value)		4.89 (.087)	0.31 (.579)	11.67 (.0006)	471.95 (<.0001)	224.57 (<.0001)

*Note*. All predictor variables standardized to have *M* *=* 0 and *SD* = 1. Statistically significant beta coefficients are bolded. PCV: proportion change in variance; *R*^2^m: marginal *R*^2^; *R*^2^c: conditional *R*^2^; AIC: Akaike information criterion.

* Intercept not standardized.

Overall, after SSA, the best single predictor was pulse clarity, followed by a subjective emotional response to music. The conditional *R*^2^ of the full model was ~30%, meaning that around a third of the variance in the experienced autobiographical saliency was explained by a combination of our fixed predictors (SSA, emotions, and musical features) and the differences in the overall level of autobiographical saliency of music between different persons (random factor).

### Comparing the musical features of the songs with low/high SSA and the autobiographical saliency

Here, we tested our hypothesis that the songs with low SSA/high autobiographical saliency would have more similarity to the songs with high SSA/high autobiographical saliency than to the songs with high SSA/low autobiographical saliency. Based on the visual inspection of Figure 2 and the results of Jakubowski et al. (2020), we categorized the SSA range of the songs individually for each participant using a cut-off of 20 years, to low SSA (⩽ 20 years) or high SSA (> 20 years). Similarly, the autobiographical saliency of the songs was classified individually for each participant as low (score ⩽ 3) or high (score > 3). First, we tested if an interaction between low/high SSA and low/high autobiographical saliency was present. Second, we inspected the means and confidence intervals of low/high SSA/autobiographical saliency categories to see the relative differences between them. In the domains of spectral centroid and pulse clarity, the statistical interaction was clearly present, AIC 123,491 -> 123,367; χ^2^(1) = 126.53, *p* < .001 and AIC 6,270.5 -> 6,178.7; χ^2^(1) = 93.84, *p* < .001, respectively. For key clarity, the interaction was not statistically significant, AIC -17054 -> -17055; χ^2^(1) = 2.83, *p* = .093.

**Figure 3.**
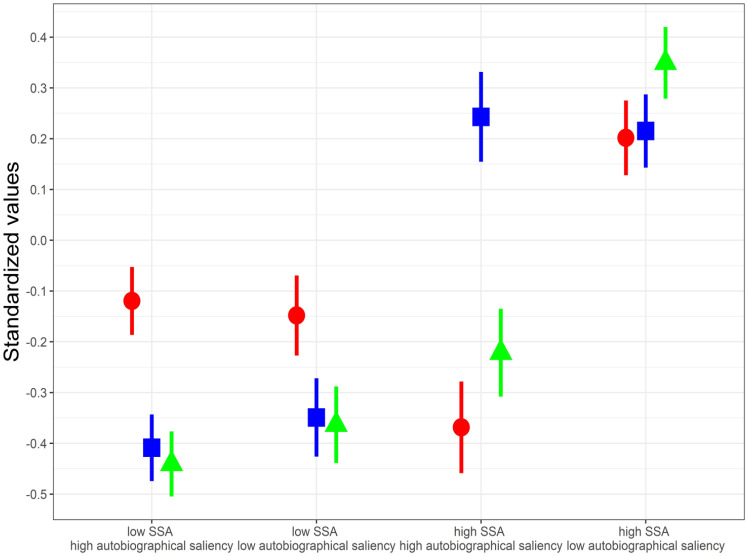
Standardized Values with 95% Confidence Intervals for Spectral Centroid (Circle), Key Clarity (Square), and Pulse Clarity (Triangle) in Different SSA and Autobiographical Saliency Groups.

When inspecting Figure 3 and Table 3, we see that for the songs from participants’ youth (low SSA), all musical features were relatively independent of the song’s average autobiographical saliency. In contrast, for the later songs (high SSA), there were clear differences between high and low autobiographical saliency in spectral centroid and pulse clarity. For pulse clarity, in line with our hypothesis, it is clear that the songs from the participants’ youth were more similar to the later songs with high autobiographical saliency than to the songs with low autobiographical saliency. This means that later and less autobiographically salient songs have a clearer pulse on average than the rest of the songs in our sample. As an example, one can imagine that songs from the 50s or the 60s typically had less clear beat, and people who were young in that era possibly grew to like such music, and therefore also later music with less clear beat could have a higher probability of being personally salient to them. For spectral centroid, this distinction was more complex: While the later songs with low autobiographical saliency still differed more from the songs from participants’ youth, the later songs with high autobiographical saliency also showed a clear difference, but in the opposite direction, having a lower centroid in the frequency spectrum on average. Finally, key clarity showed distinctly only an effect of time, where the songs from participants’ youth had less clear key clarity on average than the later songs.

**Table 3.** Estimates of the Musical Features in Different SSA/Autobiographical Saliency Groups.

	*n*	Spectral centroid^a^ [95% CI]	Key clarity^b^ [95% CI]	Pulse clarity^b^ [95% CI]
Low SSA High autobiographical saliency	820	2,225.20 [2,181.97, 2,268.43]	0.584 [0.578, 0.590]	−1.475 [−1.500, −1.451]
Low SSA Low autobiographical saliency	2,131	2,206.66 [2,155.25, 2,257.53]	0.589 [0.583, 0.596]	−1.445 [−1.474, −1.417]
High SSA High autobiographical saliency	1,002	2,064.34 [2,006.05, 2,122.63]	0.639 [0.632, 0.647]	−1.391 [−1.424, −1.358]
High SSA Low autobiographical saliency	3,887	2,432.78 [2,385.21, 2,480.35]	0.637 [0.631, 0.643]	−1.172 [−1.200, −1.145]

*Note*. SSA: song-specific age; CI: confidence interval.

a Measured in Hertz.

b Measured in arbitrary units.

## Discussion

We have presented three main findings concerning older adults: (1) The newer songs that are more autobiographically salient are more similar in rhythmic aspects to the songs from listeners’ youth, when compared to less autobiographically salient newer songs; (2) Participants’ overall emotional responsiveness to music may predict their average autobiographical saliency ratings of music to some degree; (3) Tonal, rhythmic, and timbral aspects of music had some predictive value for autobiographical saliency after accounting for effects of emotional responsiveness and SSA. Based on our results, we can also conclude that age, gender, and subjective memory functioning do not play a central role in the overall autobiographical saliency of music, at least in healthy people over 65 years old.

The relationship between musical features and autobiographical memories has already been established by Salakka et al. (2021). However, the approach in that study was exploratory in nature, and it utilized principal components as predictors to balance the number of predictors and explanatory power. In the present study, we chose a priori three musical features representing central elements of music: spectral centroid (timbre), key clarity (tonality), and pulse clarity (rhythm). All these elements were able to explain some parts of the autobiographical saliency in music after adjusting for age, gender, subjective memory, emotional response to music, and SSA. This contributes to the idea that, at least to some degree and for a certain age range of listeners (in this case, over 65 years), it is possible to predict a song’s autobiographical saliency from the music itself. Of the three musical features used here, pulse clarity had the strongest connection to autobiographical saliency, reflecting the relative importance of rhythm in musical memory processes (consolidation or recall). Since the strong connection between musical emotions and musical memories is known, this finding might also reflect the mediative role of the limbic system between rhythmic and emotional processing of music (Alluri et al., 2012; Singer et al., 2016). Prior studies have also advocated the importance of pulse clarity in emotional ratings of music, although not always over spectral features (Eerola, 2011; Gingras et al., 2014; Luck et al., 2008; Salakka et al., 2021). It is possible that through this emotional link, rhythm might have some effect on the autobiographical saliency of music.

We also replicated the finding that the reminiscence bump predicts the relationship between listeners’ SSA and autobiographical saliency. The bump was observed in a similar time period with previous studies (Jakubowski et al., 2020; Janssen et al., 2005; Platz et al., 2015; Schulkind et al., 1999; Schulkind & Woldorf, 2005), peaking around teenage years, but occurred a little earlier than usual (11 years in our study vs. around 15 years on average). Our study used a sample of people over 60 years old, which might have affected the location of the bump. It is also possible that, at least for some participants, the personal events related to the song happened later than during the year it was popular. However, from Figure 1, we can see that the large variability around the fitted function and a relatively small marginal *R*^2^ value indicate that this function predicts only a small part of the variability in the autobiographical saliency of the songs. While the cubic function was clearly a better predictor than its quadratic or linear counterparts by statistical tests, the difference between these functions was not large in terms of effect size. Practically this implies that while it is better to use the cubic function when reasonable, the linear function might work almost as well, especially with a similar SSA range to the present study and in the presence of high variance. The slight increase in the autobiographical saliency for an SSA of more than 45 might represent a small recency effect, although it might also be just a sampling artifact due to the small number of participants who were old enough to have an SSA over 45 for the 80s songs.

Higher subjective emotional responsiveness to music was related to higher average autobiographical saliency of the songs, indicating that people who used music as a means of mood regulation or tended to express more emotions while listening to music tended to feel the music as more autobiographically important on average. The effect of emotional responsiveness was not large, but small effect sizes are typical when investigating individual differences (Gignac & Szodorai, 2016). From earlier research, we already know that songs that evoke strong emotions tend to evoke strong memories (Schulkind et al., 1999; Schulkind & Woldorf, 2005). Based on the present study, we can extend this by saying that emotional overall responsiveness to music may lead to more autobiographically salient musical memories. Subjective memory functioning, in turn, did not show a statistical relationship to the autobiographical saliency of music. This finding was not particularly surprising, given that our participants were generally healthy older people and that musical memory can deteriorate relatively slowly even in neurodegenerative diseases (Cuddy & Duffin, 2005; Cuddy et al., 2012; El Haj, Postal, & Allain, 2012; Vanstone & Cuddy, 2009b). This result, however, gives us a hint that the autobiographical saliency of music might be unaffected by possible memory deficits related to healthy aging.

For all of these three factors (musical features, SSA, and emotional responsiveness), it is also important to consider if their effects were seen in between-participant variance or in residual variance. In other words, do they reflect differences between people, or are they more consistent across people? Emotional responsiveness is seen as an attribute of the participant and, therefore, obviously reflects individual differences between the participants. Importantly, the participants also could have had individual differences regarding the effects of musical features and SSA, but this was not seen in our results. Instead, our results suggested that, on average, the musical features and SSA affected the autobiographical saliency in a more consistent manner, but because of still high residual variance, the predictive power of these factors remained relatively low.

Based on former research (Salakka et al., 2021), we hypothesized that newer songs that are musically similar to older songs are also experienced as more autobiographically salient than songs having a different musical profile. Our hypothesis was statistically confirmed in the domain of rhythm with the finding that the newer autobiographically salient songs were actually more similar to the older songs in general (and not just to the autobiographically salient older songs). In other words, the rhythmic distance (measured by pulse clarity) between autobiographically salient and less salient songs depended on SSA. This finding is particularly interesting because it strengthens the conclusion that the musical style of the song can itself (directly or indirectly) contribute to the formation or recall of musical memories. Concerning timbre, our results suggest that when it comes to newer songs, older adults might value certain timbral characteristics (low spectral centroid) even more than in older songs. In tonal domain, we observed just the effect of song age, with newer songs having clearer tonal structure than older songs.

We found no effect for the demographic factors of age and gender. For age, our results are in line with Janssen et al. (2005), who found no effect of age for the autobiographical reminiscence bumps. However, they found that gender affects the location of the reminiscence bump for people from the USA but not for Dutch people. In turn, in their explorative study, Jakubowski and Ghosh (2021) did not find any relationship between gender and the autobiographical saliency ratings of music, but small to moderate correlations between age and vividness/emotionality of music-evoked autobiographical memories. Overall, when the results of these studies are combined, including the present one, it seems that the listener’s age and gender have little or no relationship to their ratings of the autobiographical saliency of music.

Concerning the limitations of this study, the small variance of the subjective memory (PRMQ) scores in our sample limits the conclusions that can be drawn. Most of the participants did not report notable problems in memory functioning, which leaves us with an open question of whether people with significant memory difficulties might show different results. As SSA was based on the popularity year of the song, this might have had a small biasing effect on the location of the reminiscence bump. However, as mentioned above, the location of the reminiscence bump is in fair agreement with former results and thus should not be of concern (e.g., Janssen et al., 2005). The sample of this study consisted only of older adults, which limits the generalizability of the results to younger people, but on the other hand, and it has more predictive power within this specific age range. With a wider age range of participants and songs, it would be possible to examine the effect of SSA on autobiographical saliency more extensively. Also, because our song pool was limited to music from the 1950s to the 1980s, the maximum SSA for some of our participants was quite low (33 years). However, the age of our average participant was 10 years higher, and our analysis method (LMM) takes into account individual variation, so this should not pose a major problem. Worth mention is also the fact that the sample of this study consisted of large number of choir singers relative to the general population. Lastly, while not a major limitation, we are aware that the English translation of the question about autobiographical saliency might look more like a frequency measure than a measure of strength or intensity of memories. This issue arises mostly from the translation, and its Finnish counterpart is not interpreted as strictly as a frequency measure, but in a way, how “memory-evoking” the song is.

In conclusion, our results indicate that SSA (especially when accounting for the reminiscence bump) and timbral, tonal, and rhythmic profile of music have an effect on the experienced autobiographical saliency of songs in older adults. The effects regarding musical features were quite small, but they showed that single features could have a statistical connection to the autobiographical saliency of a song. In addition, the reminiscence bump puts more weight on the musical memories formed in adolescence. This was evident in our study, especially in the domain of rhythm (and possibly timbre), where we observed that the musical distance between high and low autobiographical saliency depended on whether the song was popular in participants’ youth or during later life. Finally, higher emotional responsiveness to music potentially increases the overall level of experienced autobiographical saliency of music. Overall, to a limited degree, older people with high emotional responsiveness to music have the most potential to feel the music as autobiographically salient, especially when the song has a less clear pulse and was popular in their youth.

## Supplemental Material

sj-jpg-3-pom-10.1177_03057356231186961 – Supplemental material for Emotional and musical factors combined with song-specific age predict the subjective autobiographical saliency of music in older adults Supplemental material, sj-jpg-3-pom-10.1177_03057356231186961 for Emotional and musical factors combined with song-specific age predict the subjective autobiographical saliency of music in older adults by Ilja Salakka, Anni Pitkäniemi, Emmi Pentikäinen, Pasi Saari, Petri Toiviainen and Teppo Särkämö in Psychology of Music

sj-pdf-1-pom-10.1177_03057356231186961 – Supplemental material for Emotional and musical factors combined with song-specific age predict the subjective autobiographical saliency of music in older adults Supplemental material, sj-pdf-1-pom-10.1177_03057356231186961 for Emotional and musical factors combined with song-specific age predict the subjective autobiographical saliency of music in older adults by Ilja Salakka, Anni Pitkäniemi, Emmi Pentikäinen, Pasi Saari, Petri Toiviainen and Teppo Särkämö in Psychology of Music

sj-pdf-2-pom-10.1177_03057356231186961 – Supplemental material for Emotional and musical factors combined with song-specific age predict the subjective autobiographical saliency of music in older adults Supplemental material, sj-pdf-2-pom-10.1177_03057356231186961 for Emotional and musical factors combined with song-specific age predict the subjective autobiographical saliency of music in older adults by Ilja Salakka, Anni Pitkäniemi, Emmi Pentikäinen, Pasi Saari, Petri Toiviainen and Teppo Särkämö in Psychology of Music
