# Supplementary figures and images for: Emotional and musical factors combined with song-specific age predict the subjective autobiographical saliency of music in older adults
Source: Psychol Music. 2023 Oct 16;52(3):305–21. doi: 10.1177/03057356231186961 (PMC11068497; doi:10.1177/03057356231186961)

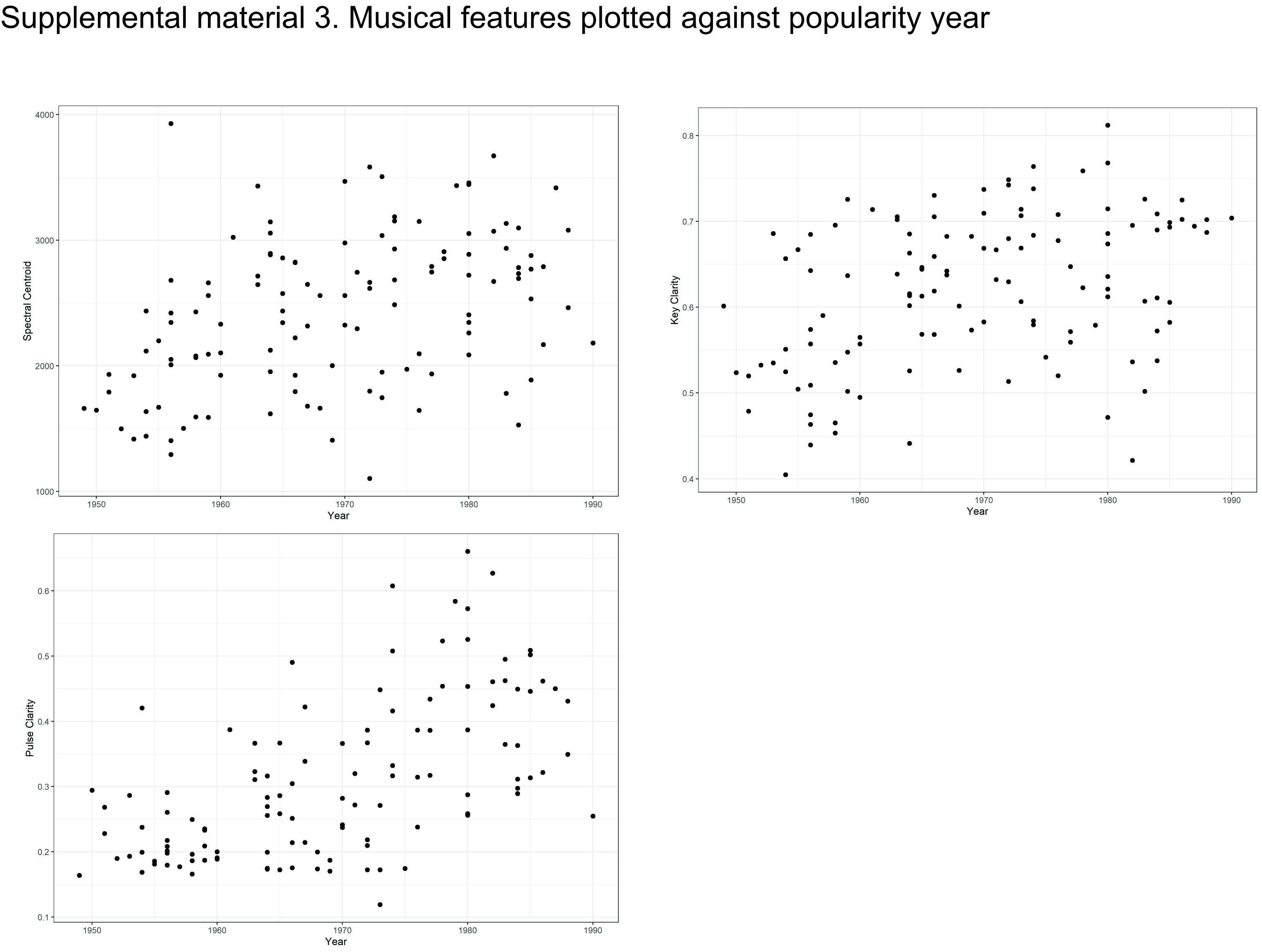

Supplement: sj-jpg-3-pom-10.1177_03057356231186961 – Supplemental material for Emotional and musical factors combined with song-specific age predict the subjective autobiographical saliency of music in older adults [file sj-jpg-3-pom-10.1177_03057356231186961.jpg]
