# Supplementary material for: Emotional and musical factors combined with song-specific age predict the subjective autobiographical saliency of music in older adults
Source: Psychol Music. 2023 Oct 16;52(3):305–21. doi: 10.1177/03057356231186961 (PMC11068497; doi:10.1177/03057356231186961)
Supplement: sj-pdf-1-pom-10.1177_03057356231186961 – Supplemental material for Emotional and musical factors combined with song-specific age predict the subjective autobiographical saliency of music in older adults [file sj-pdf-1-pom-10.1177_03057356231186961.pdf]

Supplemental material 1. Forming of the song lists

The archives of main Finnish radio channels were searched to identify a total of 225 songs comprising music from the 1950s to the 1980s (popular, rock, and jazz music) and traditional folk songs. The language of all songs was either Finnish or English. This song pool formed the musical stimuli of the pilot experiment (n = 11) that was carried out to exclude the songs with extremely high or low average familiarity and autobiographical saliency from final song pool. The final song pool consisted of 140 songs total (Table S1) divided to two lists with relatively balanced song familiarity and autobiographical saliency (A and B, 70 songs in each), matched for genre and era. Both lists comprised 10 folk songs and 15 songs from each four decades (1950s–1980s). The participants were allocated into two groups (corresponding to A and B song lists) in a way that both groups matched for age, gender, and choir singing background. This was done to include large number of songs without making the listening task too tiresome for an individual participant. For the present study, folk songs were removed from the data because computing individual song-specific age for them was not possible.

Table S1. Full list of the 140 songs used in the study.

Song genre	Artist	Song title
**1950s popular songs (30)**	Pirkko Jaakkola	*Pariisin taivaan alla*
	Laila Kinnunen	*Lazzarella*
	Eila Pienimäki	*Vanhan veräjän luona*
	Kipparikvartetti	*Kaunis Veera*
	Olavi Virta	*Sokeripala*
	Juha Eirto	*Tiikerihai*
	Eero Väre	*Kultainen nuoruus*
	Lasse Liemola	*Anna pois*
	Doris Day	*Whatever Will Be, Will Be (Que Sera, Sera)*
	The Platters	*Smoke Gets in Your Eyes*
	Little Richard	*Long Tall Sally*
	Harry Belafonte	*Day-O (The Banana Boat Song)*
	Nat King Cole	*Quizás, quizás, quizás*
	Louis Armstrong	*Mack the Knife*
	Chris Barber	*Petite Fleur*
	Metro-tytöt	*Orvokkeja äidille*
	Helena Siltala	*Ranskalaiset korot*
	Annikki Tähti	*Kuningaskobra*
	Brita Koivunen	*Suklaasydän*
	Tapio Rautavaara	*Isoisän olkihattu*
	Kauko Käyhkö	*Rovaniemen markkinoilla*
	Georg Ots	*Saarenmaan valssi*
	Veikko Tuomi	*Vanhan vaahteran laulu*
	Rosemary Clooney	*Mambo italiano*
	Pat Boone	*Love Letters in the Sand*
	Bill Haley	*Rock Around the Clock*
	Elvis Presley	*Heartbreak hotel*
	The Four Lads	*Istanbul (Not Constantinople)*
	Louis Prima	*Buona Sera*
	Glenn Miller	*Moonlight Serenade*
**1960s popular songs (30)**	Tamara Lund	*Sinun omasi*
	Katri Helena	*Minne tuuli kuljettaa*
	Reijo Taipale	*Tähdet meren yllä*
	Mauno Kuusisto	*Kertokaa se hänelle*
	Danny	*Piilopaikka*
	Kari Kuuva	*Tango pelargonia*
	Four Cats	*Suuret setelit*

	Dusty Springfield	*You Don't Have to Say You Love Me*
	Billy J. Kramer & The Dakotas	*Bad to Me*
	The Monkees	*I'm a Believer*
	The Rolling Stones	*The Last Time*
	The Animals	*House of the Rising Sun*
	The Swinging Blue Jeans	*Hippy Hippy Shake*
	Stan Getz & João Gilberto	*The Girl from Ipanema*
	Ray Charles	*Hit the Road Jack*
	Vieno Kekkonen	*Ei koskaan sunnuntaisin*
	Pirkko Mannola	*Kuinka rakkaus alkoi*
	Pasi Kaunisto & Nacke Johansson's Orchestra	*Koskaan et muuttua saa*
	Eino Grön	*Sä kuulut päivään jokaiseen*
	Aikamiehet	*Iltatuulen viesti*
	Johnny	*Hyvin menee kuitenkin*
	Eero Raittinen	*Vanha holvikirkko*
	The Sounds	*Emma*
	The Beatles	*All My Loving*
	Tom Jones	*Delilah*
	Simon & Garfunkel	*Bridge Over Troubled Water*
	Procol Harum	*A Whiter Shade of Pale*
	The Beach Boys	*Good Vibrations*
	The Renegades	*Cadillac*
	Aretha Franklin	*Chain of Fools*
**1970s popular songs (30)**	Vicky Rosti	*Tuolta saapuu Charlie Brown*
	Merja Rantamäki	*Mistä mä löytäisin sen laulun*
	Jukka Kuoppamäki	*Kultaa tai kunniaa*
	Jussi & the Boys	*Metsämökin tonttu*
	Deep Purple	*Black Night*
	Hector	*Olen hautausmaa*
	Jamppa Tuominen	*Aamu toi, ilta vei*
	Irwin Goodman	*St. Pauli ja Reperbahn*
	ABBA	*Waterloo*
	Lynn Anderson	*Rose Garden*
	Elton John	*Crocodile Rock*
	The Rubettes	*Sugar Baby Love*
	Creedence Clearwater Revival	*Travelin' Band*
	Donna Summer	*Hot Stuff*
	Carl Douglas	*Kung Fu Fighting*
	Katri Helena	*Syysunelma*
	Fredi	*Puhu hiljaa rakkaudesta*
	Erkki Junkkarinen	*Ruusuja hopeamaljassa*
	Kai Hyttinen	*Dirlanda*
	Leevi & the Leavings	*Mitä kuuluu, Marja-Leena?*
	Tuomari Nurmio	*Valo yössä*
	Hurriganes	*Get on*
	Kontra	*Jerry Cotton*
	Baccara	*Yes Sir, I Can Boogie*
	Middle of the Road	*Chirpy Chirpy Cheep Cheep*
	Uriah Heep	*Lady in Black*
	Christie	*Yellow River*
	Led Zeppelin	*Whole Lotta Love*
	Roberta Flack	*Killing Me Softly with His Song*
	Boney M.	*Rivers of Babylon*
**1980s popular songs (30)**	Paula Koivuniemi	*Tummat silmät, ruskea tukka*

	Vera Telenius	*Miljoona ruusua*
	Topi Sorsakoski	*Eeva*
	Kirka	*Surun pyyhit silmistäni pois*
	Pirkka-Pekka Petelius	*Muistan sua Elaine*
	Miljoonasade	*Marraskuu*
	Leevi & the Leavings	*Pohjois-Karjala*
	Pelle Miljoona	*Moottoritie on kuuma*
	Barbara Streisand	*Woman in Love*
	Madonna	*Papa Don't Preach*
	Toto	*Africa*
	David Bowie	*Let's Dance*
	Earth, Wind & Fire	*Celebration*
	Marvin Gaye	*I Heard It through the Grapevine*
	Diana Ross	*Upside Down*
	Tuula Amberla	*Lulu*
	Lea Laurila	*Ei oo, ei tuu*
	Matti & Teppo	*Mä näitä polkuja tallaan*
	Rauli Badding Somerjoki	*Tähdet tähdet*
	Juice Leskinen	*Kaksoiselämää*
	J. Karjalainen ja Mustat Lasit	*Doris*
	Juha Vainio ja Hyvän Tuulen Laulajat	*Albatrossi*
	Eppu Normaali	*Kitara, taivas ja tähdet*
	Blondie	*Call Me*
	Michael Jackson	*Billie Jean*
	Tina Turner	*Typical Male*
	Jari Huhtasalo	*Äideistä parhain*
	Leonard Cohen	*Dance Me to the End of Love*
	Ottawan	*Hands Up*
	Stevie Wonder	*I Just Called to Say I Love You*
**Traditional folk songs (20)**	(Trad.)	*Tuoll' on mun kultani*
	(Trad.)	*Kalliolle kukkulalle*
	(Trad.)	*Tuonne taakse metsämaan*
	(Trad.)	*Kotimaani ompi Suomi*
	(Trad.)	*Yksi ruusu on kasvanut laaksossa*
	(Trad.)	*Leivo*
	(Trad.)	*Säkkijärven polkka*
	(Trad.)	*Tulatullallaa*
	(Trad.)	*On neidolla punapaula*
	(Trad.)	*Laulu Suomessa*
	(Trad.)	*Taivas on sininen ja valkoinen*
	(Trad.)	*Soittajapaimen*
	(Trad.)	*Kotini*
	(Trad.)	*Lapsuuden toverille*
	(Trad.)	*Sunnuntaiaamuna*
	(Trad.)	*Jos sais kerran reissullansa*
	(Trad.)	*Minun kultani kaunis on*
	(Trad.)	*Täällä yksinäni laulelen*
	(Trad.)	*On suuri sun rantas autius*
	(Trad.)	*Heili Karjalasta*
