# Supplementary material for: Emotional and musical factors combined with song-specific age predict the subjective autobiographical saliency of music in older adults
Source: Psychol Music. 2023 Oct 16;52(3):305–21. doi: 10.1177/03057356231186961 (PMC11068497; doi:10.1177/03057356231186961)
Supplement: sj-pdf-2-pom-10.1177_03057356231186961 – Supplemental material for Emotional and musical factors combined with song-specific age predict the subjective autobiographical saliency of music in older adults [file sj-pdf-2-pom-10.1177_03057356231186961.pdf]

Supplemental material 2. VIF-values for the predictors in Table 2

Table S2. VIF-values for the predictors of the full model in Table 2

Variable	VIF
Age	1.078
Gender	1.037
PRMQ	1.059
Emotion	1.082
SSA	50.928
SSA^2	252.104
SSA^3	95.470
Spectral centroid	1.465
Key clarity	1.209
Pulse clarity	1.910
